# Data-driven estimates of global nitrous oxide emissions from croplands

**DOI:** 10.1093/nsr/nwz087

**Published:** 2019-07-11

**Authors:** Qihui Wang, Feng Zhou, Ziyin Shang, Philippe Ciais, Wilfried Winiwarter, Robert B Jackson, Francesco N Tubiello, Greet Janssens-Maenhout, Hanqin Tian, Xiaoqing Cui, Josep G Canadell, Shilong Piao, Shu Tao

**Affiliations:** 1 Sino-France Institute of Earth Systems Science, Laboratory for Earth Surface Processes, College of Urban and Environmental Sciences, Peking University, Beijing 100871, China; 2 Laboratoire des Sciences du Climat et de l'Environnement, LSCE, CEA CNRS UVSQ, Gif sur Yvette 91191, France; 3 International Institute for Applied Systems Analysis (IIASA), Laxenburg A-2361, Austria; 4 The Institute of Environmental Engineering, University of Zielona Góra, Zielona Góra 65-417, Poland; 5 Department of Earth System Science, Stanford University, Stanford 94305, USA; 6 Statistics Division, Food and Agricultural Organization of the United Nations, Via Terme di Caracalla, Rome 00153, Italy; 7 European Commission, Joint Research Centre, Ispra 21027, Italy; 8 International Center for Climate and Global Change Research, School of Forestry and Wildlife Sciences, Auburn University, Auburn, Alabama 36849, USA; 9 Global Carbon Project, CSIRO Oceans and Atmosphere, Canberra ACT 2601, Australia

**Keywords:** nitrous oxide, agricultural soils, flux upscaling, emission factor, emission inventories, temporal trend

## Abstract

Croplands are the single largest anthropogenic source of nitrous oxide (N_2_O) globally, yet their estimates remain difficult to verify when using Tier 1 and 3 methods of the Intergovernmental Panel on Climate Change (IPCC). Here, we re-evaluate global cropland-N_2_O emissions in 1961–2014, using N-rate-dependent emission factors (EFs) upscaled from 1206 field observations in 180 global distributed sites and high-resolution N inputs disaggregated from sub-national surveys covering 15593 administrative units. Our results confirm IPCC Tier 1 default EFs for upland crops in 1990–2014, but give a ∼15% lower EF in 1961–1989 and a ∼67% larger EF for paddy rice over the full period. Associated emissions (0.82 ± 0.34 Tg N yr^–1^) are probably one-quarter lower than IPCC Tier 1 global inventories but close to Tier 3 estimates. The use of survey-based gridded N-input data contributes 58% of this emission reduction, the rest being explained by the use of observation-based non-linear EFs. We conclude that upscaling N_2_O emissions from site-level observations to global croplands provides a new benchmark for constraining IPCC Tier 1 and 3 methods. The detailed spatial distribution of emission data is expected to inform advancement towards more realistic and effective mitigation pathways.

## INTRODUCTION

Croplands are the largest anthropogenic source of atmospheric nitrous oxide (N_2_O) [1,2]. Over the last century, this source increased with N-fertilizer uses, accounting for 80% of the global increase in terrestrial N_2_O emissions [3]. Nevertheless, the quantifications of cropland-N_2_O emissions and of the underlying emission factors (EFs; defined as N_2_O–N emission per unit of fertilizers N applied) remain highly uncertain [4], primarily attributable to high spatiotemporal variability [5] and complex biotic and abiotic factors [1] that control soil N_2_O production. Global cropland-N_2_O emissions for the most recent decade estimated by various bottom-up approaches [3,6,7] ranged from 1.5 to 5.0 Tg N yr^–1^.

The use of EFs multiplied by activity data (i.e. N-fertilizers applied) is the most common bottom-up approach, corresponding to the Tier 1 IPCC methodology. This pragmatic approach is used in research studies and for compilation of national greenhouse gas emission inventories [6–8]. Tier 1 methods that assume temporally or regionally constant EFs provide a first-order approximation, which needs to be complemented by more detailed approaches to reduce estimation uncertainty at finer scales [3,9]. A global synthesis of site-level observations suggests that the response of N_2_O emissions to increasing N-application rates [10] is non-linear (implying non-constant EFs) and strongly depends on changing environmental conditions [11–14]. Evidence for non-linear characteristics of EFs has recently been confirmed regionally as well [15].

Tier 3 methods, such as process-based models, are arguably more realistic than the Tier 1 approach because they improve the bio-physical representation of processes involved N_2_O production [16,17]. However, their parameters are generally calibrated at a limited number of observation sites [4,18,19]. Another common source of systematic error associated with Tier 3 methods comes in part from uncertain gridded N-input data [20,21]. Because high-resolution, crop-specific data of N inputs are not available at regional or global scales from ground observations, a disaggregation of national-scale data is usually performed  [20–23]. These weaknesses lead to large uncertainties not only in estimating emissions over time and space, but also in identifying underlying drivers.

**Figure 1.**
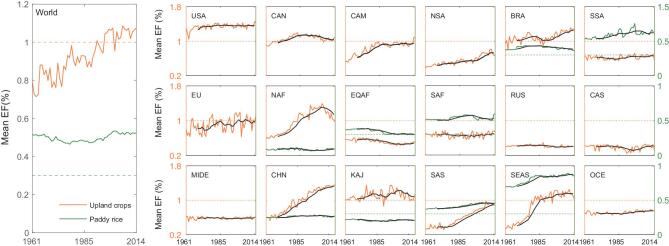
Temporal variability of cropland-N_2_O emissions globally and across 18 regions. Eighteen regions are divided consistently with the NMIP [3], including the United States (USA), Canada (CAN), Central America (CAM), Northern South America (NSA), Brazil (BRA), Southwest South America (SSA), Europe (EU), Northern Africa (NAF), Equatorial Africa (EQAF), Southern Africa (SAF), Russia (RUS), Central Asia (CAS), Middle East (MIDE), China (CHN), Korea and Japan (KAJ), South Asia (SAS), Southeast Asia (SEAS) and Oceania (OCE); solid and dashed lines indicate the EFs of our estimate and IPCC Tier 1 default; black lines indicate 10-year moving average values.

**Figure 2.**
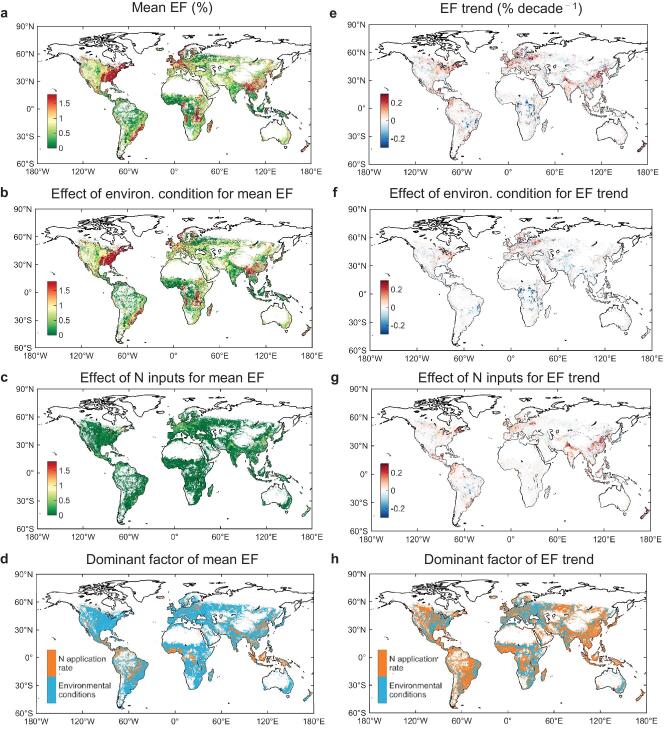
Spatial patterns of cropland-N_2_O EFs controlled by the changes in N inputs and environmental conditions. (a) and (e) Mean values and temporal trends of EFs in the period 1961–2014, respectively. (b) and (f) The effect of environmental changes on the mean and slope of EFs, respectively. (c) and (g) The effect of N-input changes on the mean and slope of EFs, respectively. (d) and (h) Dominant factor of the mean and slope of EFs, respectively, defined as the driving factor that contributes the most to the values of the mean and slope of EFs in each cropland grid cell.

To fill the gap between the simple (Tier 1) and complex (Tier 3) methods, we provide an empirical upscaling of site-level observations to quantify global cropland-N_2_O emissions. Our upscaling algorithm, a spatially referenced non-linear model [24] (SRNM), simulates N_2_O emissions incorporating the non-linear characteristics of EF and its environmental controls (see Methods). The principle is to train an algorithm to reproduce *in situ* measurements of EF at multiple sites using predictors such as climate, soil and N inputs, then produce maps of N_2_O fluxes from gridded fields of those predictors [24–27]. This approach is independent of theoretical-model assumptions (except for the choice of predictors) [28], but its performance depends on the density and representativeness of site-level observations and on the quality of gridded predictor data (e.g. N inputs). To broaden the range of environmental and management-related conditions

[5,29], we aggregate 1206 chamber-based observations of EF from 28 countries. Second, we develop a specific reconstruction of gridded N inputs disaggregated from sub-national surveys covering 15593 administrative units (see Methods).

As part of the global N_2_O budget assessment from the Global Carbon Project and the International Nitrogen Initiative [3], we present a new analysis of the global distribution and trends of cropland-N_2_O emissions in 1961–2014. We first present the spatial patterns of EFs at 5-arc-minute resolution for both upland crops and paddy rice and associated emissions results. Using sensitivity simulations (see Methods), we then attribute differences between our estimates and IPCC Tier 1 and 3 global inventories. For this analysis, we only consider direct N_2_O emissions from croplands where synthetic fertilizers, livestock manure and crop residues are added. Emissions from global permanent meadows and pastures are not considered because of a lack of site observations.

## RESULTS

### Model performance

Evaluated by cross-validation with EF data from 180 globally distributed chamber-based N_2_O flux observation sites (Supplementary Fig. 1a), our SRNM model outputs performed well, resulting in an adjusted coefficient of determination (*R*^2^_adj_) of 0.65 for upland crops (*n* = 1052, slope = 0.92, *P* <0.001, Supplementary Fig. 1b) and 0.87 for paddy rice (*n* = 154, slope = 0.91, *P* <0.001, Supplementary Fig. 1c). Our model also reproduced fairly well the long-term inter-annual variability of EFs and the sensitivity of EFs to environmental changes (*R*^2^_adj_ = 0.38-0.65, Supplementary Fig. 2). However, our EF estimates were the least well constrained for upland crops in Equatorial Africa, Southern Africa, Russia and Brazil, given the fact that the observations were relatively rare in these regions (<10% of the total, Supplementary Fig. 1d).

**Figure 3.**
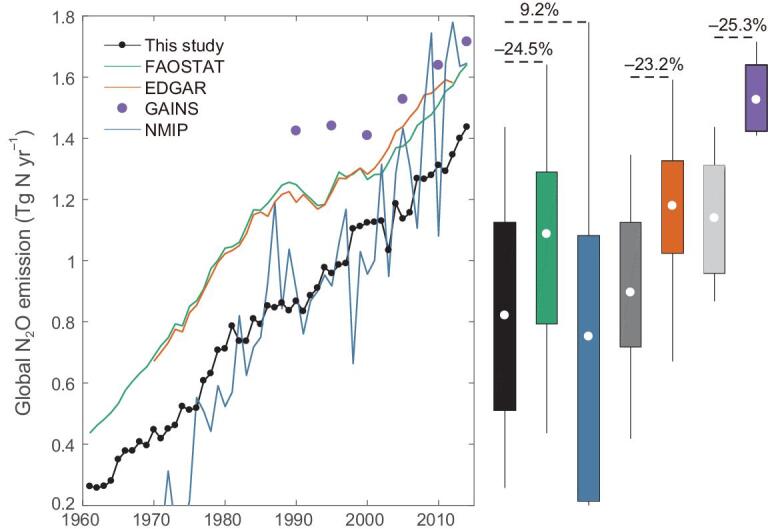
Estimates of global cropland-N_2_O emissions in 1961-2014. We normalize the FAOSTAT and GAINS by removing the contribution from synthetic fertilizers applied to pasture, the EDGAR version 4.3.2 by excluding the contributions from synthetic fertilizers applied to pasture and soil mineralization and the NMIP by excluding the contribution from ‘background’ emissions. The box plots (mean, one standard deviation and minimum-to-maximum range) are given for the period 1961-2014 for prominent datasets (colors) and for our estimates in different periods (1961-2014: black, 1970-2012: gray, 1990-2015 in 5-yr increments: light gray). The proportion value indicates the emission difference in means between our estimates and prominent datasets, computed as the difference divided by the estimates of prominent datasets.

## Emission factors

Based on our validated SRNM, reconstructed gridded N inputs, and climate and edaphic factors, our estimate suggests that global cropland-N_2_O EF for upland crops increased from 0.80 ± 0.06% in the 1960s (*σ* is the standard deviation of EFs occurring over a decade) to 1.05 ± 0.04% in the last decade (2005–14; Fig. 1). However, EFs for paddy rice remained relatively stable at between 0.46 and 0.53% over the past six decades. The SRNM model confirms IPCC Tier 1 default for upland crops in 1990–2014, but gives a lower EF before that and a larger EF for paddy rice by approximately two-thirds over the full period 1961–2014.

The substantial regional differences were further identified for both crop systems (Fig. 1). For upland crops, China (CHN), Southeast Asia (SEAS), South Asia (SAS) and North Africa (NAF) had double the growth rate in EFs than observed for the global average. Equatorial Africa, in contrast, had decreasing EFs over the past six decades. The other regions including the USA and EU had relatively constant EFs over the full period. Although most of the regions showed upland-crop EFs close to the IPCC Tier 1 default after 1990, marginal crop-producing regions had substantially smaller EFs, as much as two-thirds less than the default. For paddy rice, EFs in China, Korea and Japan (KAJ) and North Africa were very close to the IPCC Tier 1 default, but EFs in SAS and SEAS ranged from half to two times larger values, respectively.

Large spatial contrasts of EFs averaged over the period 1961–2014 are apparent in Fig. 2a, attributable primarily to differences in local environmental conditions across 77% of the global cropland area (Fig. 2b–d). This result differs from previous non-linear models [10,26,30] where spatial differences in EFs depend primarily on N-application rates. The importance of environmental controls on the non-linear characteristics of EFs was confirmed by the recent global synthesis [10] that defined the EF as EF^0^ + ΔEF × N rate, where EF^0^ is a baseline of the EF associated with climate and edaphic factors [24] and ΔEF × N the fertilizer-induced increment of an EF. For most of crop and fertilizer types, EF^0^ was found to be a more important component than ΔEF × N for N-application rates below 160 kg N ha^–1^ (i.e. approximately twice the global average; Supplementary Table 1). In contrast to the drivers of spatial gradients, a global increase in EF (0.07 ± 0.22 % decade^–1^) was mainly controlled by the growth in N-application rates covering 57% of global cropland area (Fig. 2e–h). The sensitivity of the global mean EF to increasing N addition was estimated as 0.27–0.58% per 100 kg N ha^–1^ (*μ* – *σ*, *μ* + *σ*) for upland crops and 0.01–0.07% per 100 kg N ha^–1^ for paddy rice during 1961–2014, which is comparable to several recent studies (–0.45–0.79 and –0.02–0.20% per 100 kg N ha^–1^, respectively) [10,14,31].

**Figure 4.**
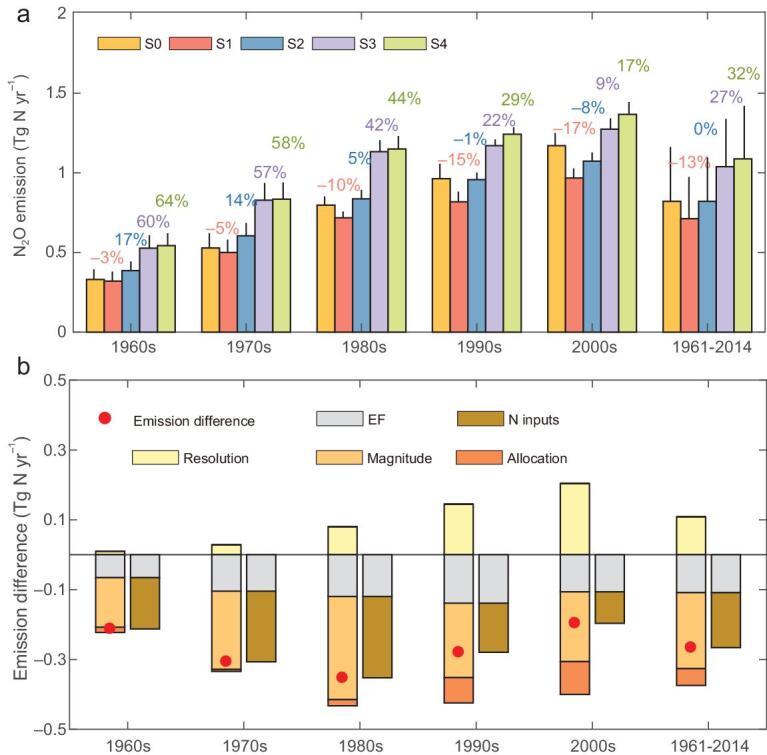
Results of scenario simulations and the attribution of emission differences globally. (a) Global cropland-N_2_O emissions of five simulations for different periods; the difference ratio is calculated as the difference between S0 and other simulations divided by S0; the scenarios S0-S4 are defined in Methods. (b) Difference between S0 and S4 due to the effects of different EFs and N inputs, i.e. the more detailed the spatial resolution of the N inputs (S0-S1), the lower the EFs (S1-S2), the lower the N inputs for croplands (S2-S3) and the revised allocation of N inputs by crop (S3-S4). The combined effects of the N inputs and the emission difference between S0 and S4 are colored as dark yellow and red in (b), respectively.

## Emission differences with Tier 1 methods

We estimated a persistent increase in global cropland-N_2_O emissions with a trend of 21.5 Gg N yr^–2^ over the past six decades (Fig. 3). Emission hotspots were located in western Europe and eastern USA in the 1960s, but shifted to eastern China, northern South Asia and southern Brazil in the most recent decade (Supplementary Fig. 3). Our estimate of global cropland-N_2_O emissions was 0.82 ± 0.34 Tg N yr^–1^ averaged during 1961–2014, with half of the emissions occurring over ∼10% of the global cropland area (Supplementary Fig. 4). This new estimate differs with the published global inventories that rely largely on the IPCC Tier 1 method, despite using normalized approaches to compare estimates limited to direct N_2_O emissions from croplands over the same periods (see Methods and Supplementary Fig. 5). For example, our estimates were about one-quarter lower than the Food and Agriculture Organization (FAOSTAT, –24% in 1961–2014) [6], the Emissions Database for Global Atmospheric Research (EDGAR version 4.3.2, –23% in 1970–2012) [7] and the Greenhouse Gas and Air Pollution Interactions and Synergies (GAINS, –25% in 1990–2015) [8] (Fig. 3).

To understand the emission differences with IPCC Tier 1 global inventories, we conducted sensitivity tests with the SRNM model to isolate the contributions of different EFs and N inputs (Fig. 4 with the details in Methods). Globally, our estimate of cropland-N_2_O emissions (S0) ranged from 0.33 Tg N yr^–1^ in the 1960s to 1.17 Tg N yr^–1^ in the 2000s. Low-resolution cropland N inputs aggregated from our sub-national statistics (S1) resulted in smaller emissions than S0 by –3 to –17% (Fig. 4a). When using IPCC Tier 1 constant EF (S2), these underestimations were, however, totally or partially offset. The use of constant EF and FAOSTAT national data of N inputs by crop (S3) led to higher emissions than S0 by 8–60%. Such upward influence was amplified when not allocating N inputs by crop (S4, i.e. the normalized estimate of FAOSTAT), resulting in larger emissions of 17–64%. Therefore, for the full period 1961–2014, the smaller N inputs compared to those mainly employed by FAOSTAT, EDGAR and GAINS explained 82% of the reduced emissions (i.e. S0–S4; Fig. 4b), followed by the effects of the lower EFs for upland crops (42%) and the revised allocation of N inputs by crop (18%), which were nevertheless offset by the higher spatial resolution of N inputs (–42%). More specifically, before the 1990s, the misfit with IPCC Tier 1 global inventories was dominated by the downward influence from N inputs used in this study but, after that, the misfit was attributable to the lower EFs for upland crops derived from the SRNM (Fig. 4b).

**Figure 5.**
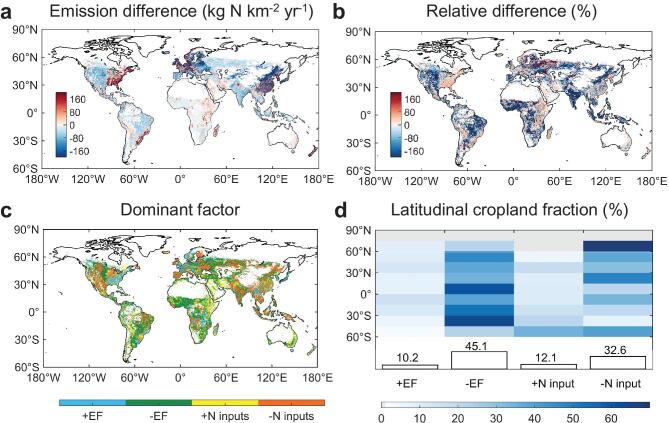
Spatial patterns of dominant drivers of the difference in cropland-N_2_O emissions. (a) Spatial distribution of the emission difference between S0 and S4 over the period 1961–2014. (b) Relative difference computed as the emission difference divided by S0. (c) Dominant factor of emission difference, defined as the driving factor that contributes the most to the difference in cropland-N_2_O emissions in each cropland grid cell. The driving factors include the updated EFs and the improved N inputs. A prefix ‘+’ of the driving factors indicates a positive effect on the emission difference, whereas ‘−’ indicates a negative effect. (d) Fractional area of croplands in latitude bands (90°N-60°S) attributed to different factors. The fraction of the cropland area (%) that is dominantly driven by each factor is labeled on top of the bar; ‘+’ and ‘−’ have the same meaning as in (c).

Emission differences are striking at the grid cell (Fig. 5a). For example, our estimate gives two times less cropland-N_2_O emissions in northern China, eastern Europe and part of central Asia (Fig. 5b). In contrast, our estimate is more than 30% larger than S4 over the top cereal-producing areas in southeast USA, eastern China and western Europe (Fig. 5b). The updated EF contributes the most to emission differences over 55% of the global cropland area (Fig. 5c and d), mainly in under-fertilized areas and the largely acidic or alkaline soils, primarily due to the non-linear characteristics of EF and its environmental controls. The revised historical N inputs (the combined effects of different quantity, distribution and allocation) dominated the rest of the croplands, particularly in Asia (Fig. 5c and d). The detailed attribution of emission differences at the gridded scale can be found in Supplementary Text 1 and Supplementary Fig. 6.

## Emission differences with Tier 3 methods

We also find emission differences between our estimate and Tier 3 modeling results from the N_2_O Model Inter-comparison Project (NMIP) [3] (Supplementary Fig. 5). In addition to uncertainties arising from model structure and parameters, another obvious reason for these differences is the scope of modeling results that represent the total N_2_O emissions from global croplands. Emission differences with Tier 3 methods may be explained by ‘background’ anthropogenic emissions [9], defined as the emissions in the absence of new fertilizer additions, including soil mineralization, atmospheric deposition on croplands derived from all sources of N as NH_3_ and NO_x_, and residual N accumulated from previous growing seasons. Thus, we estimated global ‘background’ anthropogenic emissions based on the N_2_O fluxes under unfertilized condition and FAO’s cropland-area data (see Methods), although this approach contains large uncertainties. N_2_O fluxes were estimated by the upscaling models against 469 observations for upland crops and 67 observations for paddy rice from the zero-N control sites (Adjusted *R*^2^ = 0.85 and 0.93, respectively, Supplementary Fig. 7). The estimate of historical ‘background’ anthropogenic emission is 1.52 ± 0.16 Tg N yr^–1^ (*σ* is the standard deviation representing inter-annual variability of EF) and falls within the range of previous studies (1.41–1.61 Tg N yr^–1^) [10,32] over the same period (Supplementary Fig. 7c). When removing this term, our estimate was generally consistent with NMIP results (0.82 ± 0.34 in our study; 0.75 ± 0.53 Tg N yr^–1^ in NMIP). In addition, large emission differences between our estimate and NMIP results can be found in southeast USA and most of India (Supplementary Fig. 3 vs. Fig. 4 of Tian *et al.* [3] or Fig. 6 of Tian *et al.* [4]), possibly due to the different N-input data used or different sensitivity of N_2_O flux to N inputs and environmental conditions.

## DISCUSSION

These data-driven results highlight the need for accurate estimates of global cropland-N_2_O emissions using observation-based non-linear EF and survey-based gridded N-input data. Hence, flux-upscaling models could act as a complementary approach for simple methods to estimate emissions (Tier 1 in IPCC) and for complex biogeochemical modeling with incomplete calibration (Tier 3 in IPCC). The detailed spatial resolution of our emission data will inform climate-mitigation-policy development and advancement towards a more accurate global N_2_O budget. For this reason, our analysis (i) distinguishes between the two major cropping systems, (ii) utilizes globally distributed cropland-N_2_O observations to constrain flux upscaling models and (iii) reconstructs the gridded N-input dataset of all fertilizer types where synthetic fertilizers data have been disaggregated from sub-national surveys.

Although an estimated systematic reduction (∼25%) of global cropland-N_2_O emissions is found compared to IPCC Tier 1 global inventories, we acknowledge that actual cropland-N_2_O emissions remain poorly quantified. Further work is needed to determine how much more reliable our estimates are than those of widely used inventories based on the IPCC Tier 1 defaults of EF and FAOSTAT aggregated data of N inputs. First, we suggest that data-driven non-linear EFs are more realistic than IPCC Tier 1 default values, being supported by a broad observation-based dataset across contrasting environmental conditions (Supplementary Fig. 8). The effect of lower EFs on global cropland-N_2_O emissions is consistent with the value reported in Gerber *et al.* [26] for the year 2000 (–0.08 vs. –0.11 Tg N yr^–1^). Yet, our estimate of EFs contains uncertainties due to the scarcity of observations in eastern Europe, south Asia, Russia, central Asia and the Middle East (Supplementary Fig. 1a). Together, these regions contribute ∼20% of global cropland-N_2_O emissions. Another limitation is the fact that the SRNM model does not include specific cropland-management practices [33–35] (e.g. irrigation technology, tillage and straw management) responsible for cropland-N_2_O fluxes. Besides, the quantification of EF depends also on the form of a non-linear model and the choice of predictors.

Second, our high-resolution, crop-specific data of N inputs is probably more realistic than FAOSTAT national data, as it is based on sub-national statistics of synthetic fertilizers (straight and compound) that contribute ∼86% of the global N-fertilizer consumption (Supplementary Fig. 9). However, there is significant uncertainty on how N inputs are distributed to different crop types because the sub-national data compiled did not report this information, also in the amount of N in different types, forms, timing and splitting frequency influence N_2_O fluxes. In addition, other input datasets can have large sources of uncertainties in cropland-N_2_O emissions reported here. For example, the magnitude of precipitation estimates over global land deviated by as much as 300 mm yr^–1^ among the datasets [36], particularly in complex mountain areas, northern Africa and some high-latitude regions. Temporally constant soil-attributes data used in this study may distort the dynamical evolution of cropland-N_2_O emissions.

Comparison with IPCC Tier 3 methods underscores the importance of including ‘background’ anthropogenic emissions within a global N_2_O budget. In addition to soil mineralization and indirect emissions from atmospheric deposition (due to the volatilizations from croplands and non-cropland sources), the legacy effect of fertilization is not considered well in the 2006 IPCC guidelines [3]. However, multiple pieces of evidence from over-fertilized regions (e.g. the North China Plain) [37–39] indicate significant N accumulation when the N-fertilizer application rate reaches the optimum for crop growth, triggering N_2_O emissions in subsequent years. To verify this legacy effect, process-based models could be used in the future to perform simulations with all N inputs ceased to zero abruptly and to quantify the dynamics of N_2_O emissions afterwards.

In addition, upscaling direct N_2_O emissions from site-level observations to global croplands provides an independent, yet important, dataset towards refining national greenhouse gas emission inventories submitted to the UNFCCC and towards constraining process-based models through data-model integration for the global N_2_O-budget assessment. The use of a flux-upscaling model trained by global cropland-N_2_O observations increases confidence in the global and regional estimates, although modeling results contain many uncertainties, particularly related to model structure and input forcings as well as the missing ‘background’ anthropogenic emissions. Since our direct-emission products imply that it matters when and where the input of N-fertilizer occurs, especially the change in such N inputs will have different influences on cropland-N_2_O emissions. Hence, model improvements such as those presented here are essential to adequately optimize the measures aimed at mitigating N_2_O emissions. Efficient emission reductions depend upon tackling the high-N-input areas with favorable environmental conditions for N_2_O production, given the non-linearity and environmental-mediating effects that prevail.

## METHODS

### Global cropland-N_2_O observation dataset

We aggregated cropland-N_2_O flux observations from 180 globally distributed sites from online databases, ongoing observation networks and peer-reviewed publications (Supplementary Fig. 1). Cropland mentioned here is defined as the FAO's land-use category ‘Arable land and permanent crops’, with the ‘Arable land’ component including land used for temporary crops, temporary meadows and pastures, and temporary fallow. Only chamber-based observations were included in this dataset. These data repositories are as follows: the NitroEurope, CarbonEurope, GHG-Europe (EU-FP7), GRACEnet, TRAGnet, NANORP, China-N_2_O and 14 meta-analysis datasets [10,32,40–51]. Five types of data were excluded from our analysis: (i) observations without a zero-N control for background N_2_O emission, (ii) observations from sites that used controlled-release fertilizers or nitrification inhibitors, (iii) observations not covering the entire crop-growing season, (iv) observations made in a laboratory or greenhouse and (v) observations with a minimal sampling frequency of less than one time per week. Cropland-N_2_O EFs were estimated for each non-zero-N-application rate (N) as EF = (E − E_0_)/N, where E is the observed N_2_O flux during the observation period due to the application of N inputs and other unquantified source and E_0_ is the N_2_O flux during the observation period at a zero-N control site due to other unquantified source. This yielded a global dataset of cropland-N_2_O emissions, N-rate-dependent N_2_O EFs and fertilization records from each site (i.e. 1052 estimates for upland crops from 152 sites and 154 estimates for paddy rice from 28 sites), along with site-level information on climate, soils, crop type and relevant experimental parameters. Total numbers of sites and total measurements in the dataset were more than double those for previous datasets of  N_2_O EFs (Supplementary Table 2). The extended global N_2_O-observation network covered most of the fertilized croplands, representing a broad range of environmental conditions globally (Supplementary Fig. 1).

For each site in our dataset, the variables were sorted into four broad categories: N_2_O-emissions data, climate data, soil attributes and management-related or experimental parameters. The definition and units of each factor can be found in Supplementary Table 3. N_2_O fluxes at the application rate and zero-N control within the duration of the experiment is required. The sum of cumulative precipitation and irrigation use within the measurement period was taken as a proxy for the variations in water-filled pore space [52] and the mean daily air temperature within the measurement period was used as a proxy for surface-soil temperature, because of their high correlation [53]. Soil attributes including soil pH, clay content, bulk density (BD) and soil organic carbon (SOC) were used to account for the O_2_ and available C status [54]. Management-related or experimental parameters like N and irrigation-application rates, fertilizer type, crop type, measurement frequency and duration were also collected, considering their impacts on the soil N cycling and transport in the root zone [1]. Missing values of the climate and soil factors at a few sites (i.e. <12%) were either supplemented directly by corresponding authors or taken from 1-km Harmonized World Soil Database (HWSD) v1.2 (http://www.fao.org/soils-portal/soil-survey/soil-maps-and-databases/harmonized-world-soil-database-v12/en) and CRU TS v. 3.23 (https://crudata.uea.ac.uk/cru/data/hrg/), according to latitude and longitude. Fertilization methods, irrigation technologies and tillage practices were, albeit important for determining N_2_O flux, not considered due to the lack of such information in most of current databases or publications.

## Flux upscaling model

The SRNM model [24] was applied to simulate cropland-N_2_O EFs and associated emissions. N_2_O emissions were simulated from N-application rates using a quadratic relationship, with spatially variable model parameters that depend on climate, soil properties and management practices (Equation 1). The original version of the SRNM was calibrated using field observations from China only [24]. In this study, we used the global cropland-N_2_O observation dataset to train it to create maps of gridded cropland-N_2_O EFs and the associated annual emissions at 5-arc-minute resolution from 1961 to 2014. The gridded EFs and associated emissions are simulated based on the following equations:

(1a) \begin{eqnarray*} &&{\rm EF}_{ij t}&=&{\alpha}_{ij}({x}_k){N}_{ij t} +{\beta}_{ij}({x}_k),{E}_{ij t}\nonumber\\ &&{EF}_{ij t}{N}_{ij t} +{\varepsilon}_{ij t},\kern0.5em \mathrm{where}\, \ {x}_k\in{\Omega}_i,\forall i,\nonumber\\ \end{eqnarray*}

and

(1b) \begin{eqnarray*} {\alpha}_{ij}\sim N\left({X}_k^T{\lambda}_{ij k},{\sigma}_{ij k}^2\right),{\beta}_{ij}\sim N\big({X}_k^T{\phi}_{ij k},{\sigma^{\prime}}_{ij k}^2\big), \nonumber\\ \end{eqnarray*}

(1c) \begin{eqnarray*} &&{\lambda}_{ijk} &\sim & N\left({\mu}_{ijk},{\omega}_{ijk}^2\right),{\phi}_{ijk}\sim N\big({\mu}_{ijk}^{\prime },{\omega^{\prime}}_{ijk}^2\big),\nonumber\\ &&{\varepsilon}_{ijt}\sim N\big(0,{\tau}^2\big), \end{eqnarray*}

and *i* denotes the sub-function of EFs (*i* = 1, 2, … , *I*) that applies for a sub-domain division ${\Omega}_i$ of six climate or soil factors, *j* represents the type of crop (*j* = 1–2, 1 for upland crops and 2 for paddy rice), *k* is the index of the climate or soil factors (*k* = 1–6, i.e. soil pH, clay content, SOC, BD, the sum of cumulative precipitation and irrigation, mean daily air temperature), *EF_ijt_* and *E_ijt_* denote emission factor (kg N_2_O-N (kg N)^–1^ or %) and direct N_2_O emission flux (kg N ha^–1^ yr^–1^) estimated for crop type *j* in year *t* in the *i*th type of regions, *N_ijt_* is N-application rate (kg N ha^–1^ yr^–1^) and *α* and *β* are the functions of *X_k_*. The random terms *λ* and *φ* are assumed to be independent and normally distributed, representing the sensitivity of *α* and *β* to *X_k_*. *ε* is the model error. *μ* and *μ′* are the mean effect of *X_k_* for *α* and *β*, respectively. *σ*, *σ′*, *ω*, *ω′* and*τ* are standard deviations. Optimal sub-domain division, associated parameters mean values and standard deviations were determined by using the Bayesian Recursive Regression Tree version 2 (BRRT v2) [24,25,27], constrained by the extended global cropland-N_2_O-observation dataset. The detailed methodological approach of the BRRT v2 is described by Zhou *et al.* [24].

## Gridded input datasets

The updated SRNM model was driven by many input datasets, including climate, soil properties, N inputs (e.g. synthetic N-fertilizer, livestock manure and crop residues applied to cropland), irrigation uses and the historical distribution of croplands. Cumulative precipitation and mean daily air temperature over the growing season were acquired from the CRU TS v3.23 climate dataset [55] (0.5-degree resolution), where the growing season in each grid cell was identified as the period between the planting and harvesting dates obtained from Sacks *et al.* [56]. The patterns of SOC, clay content, BD and soil pH were acquired from the HWSD v1.2 [57] (1-km resolution). Both climate and soil properties were re-gridded at a resolution of 5′ × 5′ using a first-order conservative interpolation [58]. The annual cropland area at 5-arc-minute resolution from 1961 to 2014 was obtained from the History Database of the Global Environment (HYDE 3.2.1) [59]. National cropland irrigation rates over the period 1961–2014 were calculated as the ratio of irrigation water use to irrigated area from AQUASTAT (http://www.fao.org/aquastat/) and were resampled into the gridded irrigation maps of HYDE 3.2.1.

High-resolution, crop-specific data of the N-application rate in 1961–2014 were specifically developed for this study. For synthetic-fertilizer applications, we first collected sub-national statistics (i.e. county, municipal, provincial or state levels) of N-fertilizer consumption of 15593 administrative units from local statistical agencies in 38 countries mostly during the period 1980–2014 (Supplementary Fig. 9). To expand the temporal coverage of sub-national statistics, we disaggregated N-fertilizer consumptions for the period 1961–80 from FAOSTAT [6] by using the corresponding sub-national allocations in the 1980s. To harmonize the extended dataset, we scaled N-fertilizer consumption up or down based on the ratios between values of the FAO and our sub-national statistics in the 1980s (note that the same scalar was applied for each administrative unit within a country). For the other 197 countries in the world, the statistics of N-fertilizer consumption were acquired only at the national scale from FAOSTAT [6] during the period 1961–2014. It should be noted that the N-fertilizer consumption values we collected represent the amounts of both cropland use and the other agricultural uses (e.g. pasture for grazing). Thus we separated synthetic-fertilizer use applied to croplands for each administrative unit based on the country-scale crop-wise proportion information [60,61], assuming the same proportion of cropland use within a certain country. In addition, a country without a crop-wise proportion adopted this information from the surrounding country. Second, the 5-arc-minute gridded data for manure applied to croplands for the period 1961–2014 were provided by Zhang *et al.* [23] and resampled into 15 790 administrative units. For crop residues applied to croplands, we downloaded the national data from FAOSTAT [6] (1961–2014) and disaggregated them into all administrative units, with the assumption of an equal rate of crop-residue application within a certain country. It should be noted that the world has experienced changes in administrative divisions through aggregation, disaggregation and name changes, such as the Union of Soviet Socialist Republics. Thus, we harmonized the temporal evolution of national and sub-national statistics to fit the latest Global Administrative Unit Layers, based on the historical trajectories summarized by the FAO GeoNetwork (http://www.fao.org/geonetwork/).

Combining three types of N inputs, we generated global maps of cropland N inputs of 15 790 administrative units for the period 1961–2014. To compute the crop-specific N-application rates, we allocated N inputs for upland crops and paddy rice based on the breakdown (or proportion) of total fertilizer use by crops from Rosas [62]. Crop-specific N-application rates (*N_ijt_*) were finally calculated as cropland N inputs in each of the administrative units divided by the associated cropland areas that were obtained from the HYDE 3.2.1. This new dataset of the N-application rate was finally resampled into grid maps at 5-arc-minute spatial resolution (Supplementary Fig. 10) following the dynamic cropland distributions of the HYDE 3.2.1. The assumption of a maximum combined synthetic + manure + crop residues N-application rate was 1000 kg N ha^–1^—larger than the previous threshold (700 kg N ha^–1^) [30] that was only applied for the sum of synthetic fertilizers and manure.

## Comparison with previous estimates

We examine the differences with previous emission datasets globally and by regions, including the FAOSTAT Emissions Database [6], EDGAR version 4.3.2 [7], GAINS [8] and NMIP results for croplands [3] (Supplementary Fig. 5). The scope, data source and estimation approach of N_2_O emissions differ among datasets (Supplementary Table 4). FAOSTAT and GAINS provided IPCC Tier 1 estimates of direct N_2_O emissions from synthetic fertilizers, manure applied to soils and crop residues applied to soils, based on the activity data from FAOSTAT [6]. This product used the data of synthetic fertilizers that include both cropland and pasture uses. EDGAR provided N_2_O emissions from the abovementioned three types of N inputs and soil mineralization, while keeping a separating focus on N-fixing crops and paddy rice. This product was generated using the EFs derived from the CAPRI modeling system [7] and N inputs from the FAO with allocation by crop based on IRRI [63]. NMIP provided the total cropland-N_2_O emissions that account for direct N_2_O emissions and ‘background’ emissions, using an ensemble of process-based models and different sources of N inputs [4].

To compare with our estimate, FAOSTAT is corrected by removing the contributions from synthetic fertilizers applied to pasture; the ratio of pasture synthetic-fertilizer application was determined based on the country-scale-proportion information from Heffer *et al.* [60] and Lassaletta *et al.* [61]. Similarly, EDGAR is corrected by removing the contributions from synthetic fertilizers applied to pasture and soil mineralization, where soil mineralization data were obtained from the FAOSTAT Emissions Database [6]. NMIP is corrected by removing the contributions from ‘background’ emissions, which were quantified as the product of the gridded N_2_O fluxes under unfertilized conditions and cropland-area data. The details of the quantification of ‘background’ emissions can be found in Supplementary Fig. 5.

## Attribution of emission differences

Based on the SRNM, we conducted five scenario simulations (S0–S4) for the period 1961–2014 to isolate the effects of different EFs and N inputs on the emissions differences. In simulation S0, global cropland-N_2_O emissions were estimated based on the gridded EFs derived from the SRNM and our high-resolution, crop-specific N-input data. Simulation S1 was the same as S0 but used low-resolution N inputs aggregated from our sub-national statistics. Simulation S2 used the same N-input data as S1 but the IPCC Tier 1 defaults of EFs. Simulation S3 used EFs of IPCC Tier 1 defaults and N inputs of the FAO where synthetic fertilizers for pasture were excluded. Note that, in 1961–2014, our total N inputs were on average 12% lower than the FAO that removes the pasture synthetic-fertilizer application, primarily due to the reduced amount of synthetic fertilizers applied (Supplementary Fig. 11). The reduced N inputs applied to croplands were primarily attributable to the lower synthetic-fertilizer inputs surveyed from sub-national data. Simulation S4 was the same as S3 but did not allocate N inputs by crop. It should be noted that S4 is the same as FAOSTAT when excluding the emissions arising from pasture synthetic-fertilizer application. Thus, differences among the five simulations represent the effects of spatial resolution of N inputs (S0–S1), the difference in EFs (S1–S2), the difference in the magnitude of N inputs (S2–S3) and the difference in the allocation of N inputs by crop (S3–S4) on emission differences, respectively.

## Drivers of EF dynamics

To separate the contributions from change in local environmental conditions and change in N inputs on cropland-N_2_O EF dynamics (i.e. mean values and temporal trends), we conducted one additional simulation S5. In this simulation, only environmental conditions (i.e. climate factors, land uses) varied from 1961 to 2014, while the N-application rates were fixed at the level of 1961. Thus, S5 represents the effect of change in environmental conditions and the difference between S0 and S5 represents the effect of change in N inputs.

## Data availability

All computer codes and global cropland N_2_O-observation dataset used in this study and global cropland-N_2_O emission data produced in this study can be provided by the corresponding author upon reasonable request.

## Supplementary Material

nwz087_Supplemental_File
