# Supplementary material for: Data-driven estimates of global nitrous oxide emissions from croplands
Source: Natl Sci Rev. 2019 Jul 11;7(2):441–52. doi: 10.1093/nsr/nwz087 (PMC8288841; doi:10.1093/nsr/nwz087)
Supplement: nwz087_Supplemental_File [file nwz087_supplemental_file.pdf]

Supplementary materials

Extended discussion

Text S1. Attribution of emission differences at the gridded scale

Tables

Table S1. Thresholds of N application rate by crop and fertilizer types

Table S2. Major differences of N_2O emission factor observations between our database and previous works.

Table S3. List of emission and environmental factors

Table S4. Simulation experiments for exploring the effects of EF and N inputs on cropland-N_2O emissions.

Figures

Figure S1. Validation of cropland-N_2O emission factors (EFs) model at the site-level and regional scales

Figure S2. Model performance of interannual variability of EF and the sensitivity of EF to environmental changes

Figure S3. Spatial patterns of N_2O emission across global croplands by decade

Figure S4. Cumulative proportions of cropland-N_2O emissions and arable land areas over the period 1961-2014

Figure S5. Temporal evolution of cropland-N_2O emissions

Figure S6. Spatial patterns of dominant drivers of the difference in cropland-N_2O emissions

Figure S7. The estimate of global 'background' emissions of N_2O

Figure S8. Comparison of probability density of soil and climate factors between our global cropland N_2O observation data and global data over all croplands.

Figure S9. Spatial resolution of N-fertilizer consumption across 235 countries.

Figure S10. Five-minute grid maps of global N application rates.

Figure S11. Comparison of global N inputs applied to croplands between our estimates and FAO.

Figure S12. Spatial patterns of cropland-N_2O EFs, soil pH, and N application rate over the period 1961-2014

Text S1. Attribution of emission differences at the gridded scale

Sensitivity simulations helped to attribute the dominant factors at the gridded cell scale for the differences to S4. Overall, the updated EFs had dominant contributions to reduced emissions over 45.1% of the global cropland area (Fig. 5d). Negative effects of the updated EFs in under-fertilized areas (Figs S6a) can be explained by the nonlinear response of N_2O emissions to N application rates[1]. Over the full period 1961-2014, two thirds of global cropland area have N application rates less than the global average (89.8 kg N ha^{-1}), resulting in lower nitrate and nitrite accumulations[2] which sustain N_2O production in the soil less strongly. Negative effect of the updated EFs in intensively-fertilized regions was primarily ascribed to the environmentally-mediated influences (Figs S6a). For example, EFs are as low as 0.4-0.8% in the North China Plain where soil pH is relatively high (>7.5, Fig. S12), although N application rates are extreme (>300 kg N ha^{-1} yr^{-1}). Both effects, low EFs and an exponential increase of emissions with excessive N input, have recently been observed experimentally in that region[3]. The updated EFs also contributed the most to the increased emissions over 10.2% of the global cropland area (Fig. 5d), including the other over-fertilized regions such as southern China and western Europe as well as the acidic soils in southeastern USA (soil pH of ~5.0, Fig. S12b).

The combined effects of the improved N inputs contributed the most to the emission differences, with emissions of other studies up to 32.6% higher or 12.1% lower for the global cropland area (Fig. 5d). Negative effects in most of eastern Europe and northern China were primarily driven by the reduced N inputs (Figs S6f and S13). Our data of N inputs including these regions were obtained from local statistics registers, whereas FAOSTAT's N inputs were derived as an annual balance of N production and net trade[4]. Over the full period 1961-2014, N inputs of the regions dominant by the reduced N inputs were ~30% lower than those of the FAO excluding pasture SN application (Figs S6d and S6f). The crop-specific allocation of N inputs contributed the most to the reduced emissions in rice-producing regions (Fig. S6e), because of the mean EF of paddy rice in these regions were only one third of that of upland crops. In addition,

modeling results produced positive effects on emission differences in the over-fertilized regions such as the North China Plain, primarily because of the improved spatial resolution of N inputs (Fig. S6c). In contrast to the national data of FAOSTAT, our global gridded cropland N inputs data was reconstructed mainly based on sub-national statistics (i.e., 14,693 county-level data, 161 municipal data, 739 provincial or state-level data). The underestimation bias incurred by our upscaling model when used with spatially aggregated N fertilizer application rate resulted in the estimates to be conservatively low. Although there are still uncertainties in our N inputs data, the use of high-resolution, crop-wise distribution of N application rates was more effective pathway to reduce the uncertainties of global and regional cropland-N_2O emissions.

Table S1. Thresholds of N application rate by crop and fertilizer types for the baseline of EF (EF^0) that is larger than fertilizer-induced increment ($\Delta EF \times$ N rate). Data are obtained from Shcherbak et al.[1] Note that number represents the number of implied EF^0 and ΔEF. Threshold is calculated as the ratio of EF^0 and ΔEF.

Crop or fertilizer type	Number	EF0 (%)	ΔEF (% kg N^{-1} ha)	Threshold of N rate (kg N ha^{-1})
		A	B	C=A/B
Non-N fixers	221	0.658	0.00181	363.5
Upland crops	121	0.649	0.00187	347.1
Rice	16	0.089	0.00092	96.7
Perennial grass/forage	41	0.776	0.00353	219.8
Synthetic	188	0.632	0.00264	239.4
Ammonium nitrate	27	0.506	0.00777	65.1
Calcium ammonium nitrate	36	0.802	0.00125	641.6
Controlled-release urea	6	0.557	0.00023	2421.7
Urea	58	0.457	0.00284	160.9
Urea ammonium nitrate	34	0.803	0.00067	1198.5
Manure	16	0.595	0.00262	227.1
Mixed	10	0.86	0.00016	5375.0

Table S2. Major differences of N_2O emission factor observations between our database and previous works

Source	Site			Record			Year			Period	
	Total	Upland	Rice	Total	Upland	Rice	Total	Upland	Rice	Upland	Rice
**This study**	**180**	**152**	**28**	**1206**	**1052**	**154**	**39**	**39**	**33**	**1976-2016**	**1979-2015**
Akiyama et al.[5]	17	0	17	113	0	113	10	0	10	N/A	1980-2002
Stehfest and Bouwman[6]	104	94	12	624	544	80	23	23	9	1977-2002	1979-2002
Kim et al.[7,8]	128	110	18	341	319	22		--	--	--	--
Shcherbak et al.[1]	43	36	7	630	577	53	33	33	14	1978-2010	1994-2007

Table S3. List of emission and environmental factors collected from each of experiments

Type	Factor	Unit	Included in model?
Emission	N_2O emission flux	kg N ha^{-1}	Yes
	Emission factor (EF)	%	Yes
Climatic factor [a]	Precipitation (P)	mm	Yes
	Mean daily air temperature (Temp)	°C	Yes
Soil property [b]	Soil organic carbon content (SOC)	%	Yes
	Soil pH	--	Yes
	Soil clay content (Clay)	%	Yes
	Bulk density (BD)	gcm^3	Yes
Management option	N application rate (N_{rate}) [c]	kg N ha^{-1}	Yes
	Irrigation rate (I)	mm	Yes
	Crop type [d]	--	Yes
	fertilizer type	--	No
Experimental parameters	Latitude	--	No
	Longitude	--	No
	Measurement frequency	time	No
	Experimental duration	day	No

[a] cumulative precipitation and mean air temperature for each observation are calculated as the total amount and mean value within experiment duration, respectively;

[b] values for topsoil, the depth of which is 0~20 cm.

[c] N_{rate} is defined as the sum amount of nitrogen applied within experiment duration, including synthetic fertilizer, manure and crop residue applied to croplands;

[d] includes paddy rice and upland crops.

Table S4. Simulation experiments for exploring the effects of EF and N inputs on cropland-N_2O emissions.

Scenario / dataset	Emission factor [a]	Data source of N inputs					
		Resolution [b]	Magnitude (SN) [c]	Magnitude (Manure)	Magnitude (CR)	Allocation [d]	Other
FAO / GAINS	Constant	Low	FAOSTAT incl. pasture	FAOSTAT	FAOSTAT	Crop-uniform	N/A
EDGAR	Constant	Low	FAOSTAT / IRRI	FAOSTAT	FAOSTAT	Crop-specific	Soil mineralization
NMIP	Dynamic	High	Lu et al.[28]	Zhang et al.[31]	N/A	Crop-uniform	Background emissions
S0	Dynamic	High	Local	Zhang et al.[31]	FAOSTAT	Crop-specific	N/A
S1	Dynamic	Low	Local	Zhang et al.[31]	FAOSTAT	Crop-specific	N/A
S2	Constant	Low	Local	Zhang et al.[31]	FAOSTAT	Crop-specific	N/A
S3	Constant	Low	FAO excl. pasture	FAOSTAT	FAOSTAT	Crop-specific	N/A
S4	Constant	Low	FAO excl. pasture	FAOSTAT	FAOSTAT	Crop-uniform	N/A
S5	N/A	High	Local (1961 [e])	Zhang et al.[31] (1961)	FAOSTAT (1961)	Crop-specific	Environmental condition varied

[a] The symbols 'Dynamic' and 'Constant' represent EFs derived by Equation 1 and from Tier 1 defaults of 2006 IPCC (1% for upland crops, 0.3% for paddy rice), respectively; [b] The symbols 'High' and 'Low' represent N inputs data from sub-national and national statistics, respectively; [c] 'Local' represents synthetic fertilizers from this study, 'FAO' incl. and excl. pasture represent the total agricultural uses of synthetic fertilizer of the FAOSTAT and the total excluding synthetic fertilizer applications for pasture, respectively; [d] 'Crop-specific' and 'Crop-uniform' represent N inputs data with allocation by crop (i.e., upland crops, paddy rice) and without allocation, respectively. [e] the '1961' represent that N application rates were fixed at the level of 1961, while environmental conditions (i.e., climate factors, land uses) varied from 1961 to 2014.

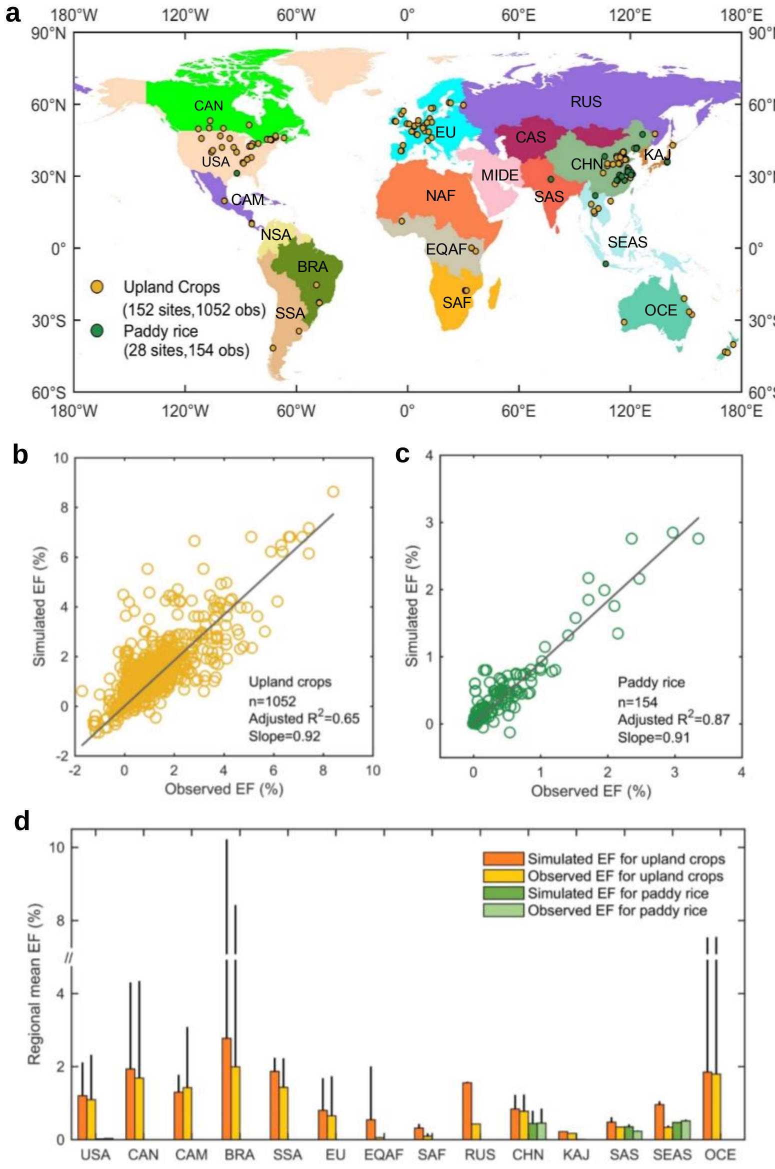

Figure S1. Validation of cropland-N_2O emission factors (EFs) model. a. Site of global cropland N_2O observation network. The 18 regions, consistent with the region boundaries of the NMIP, include United States of America (USA), Canada (CAN), Central America (CAM), Northern South America (NSA), Brazil (BRA), Southwest South America (SSA), Europe (EU), Northern Africa (NAF), Equatorial Africa (EQAF), Southern Africa (SAF), Russia (RUS), Central Asia (CAS), Middle East (MIDE), China (CHN), Korea and Japan (KAJ), South Asia (SAS), Southeast Asia (SEAS), and Oceania (OCE). **b**. Model performance of simulated EFs of upland crops; note that n, adjusted R^2, and Slope denote the number of measurements, adjusted coefficient of determination, and slope of the regression line with the intercept set to zero, respectively. **c**. Same as panel **b**, but for paddy rice. **d**. Model performance of simulated EFs for each of regions except NSA, NAF, CAS, and MIDE where no observations were collected. Error bars indicates the standard deviation of observed (or simulated) EF.

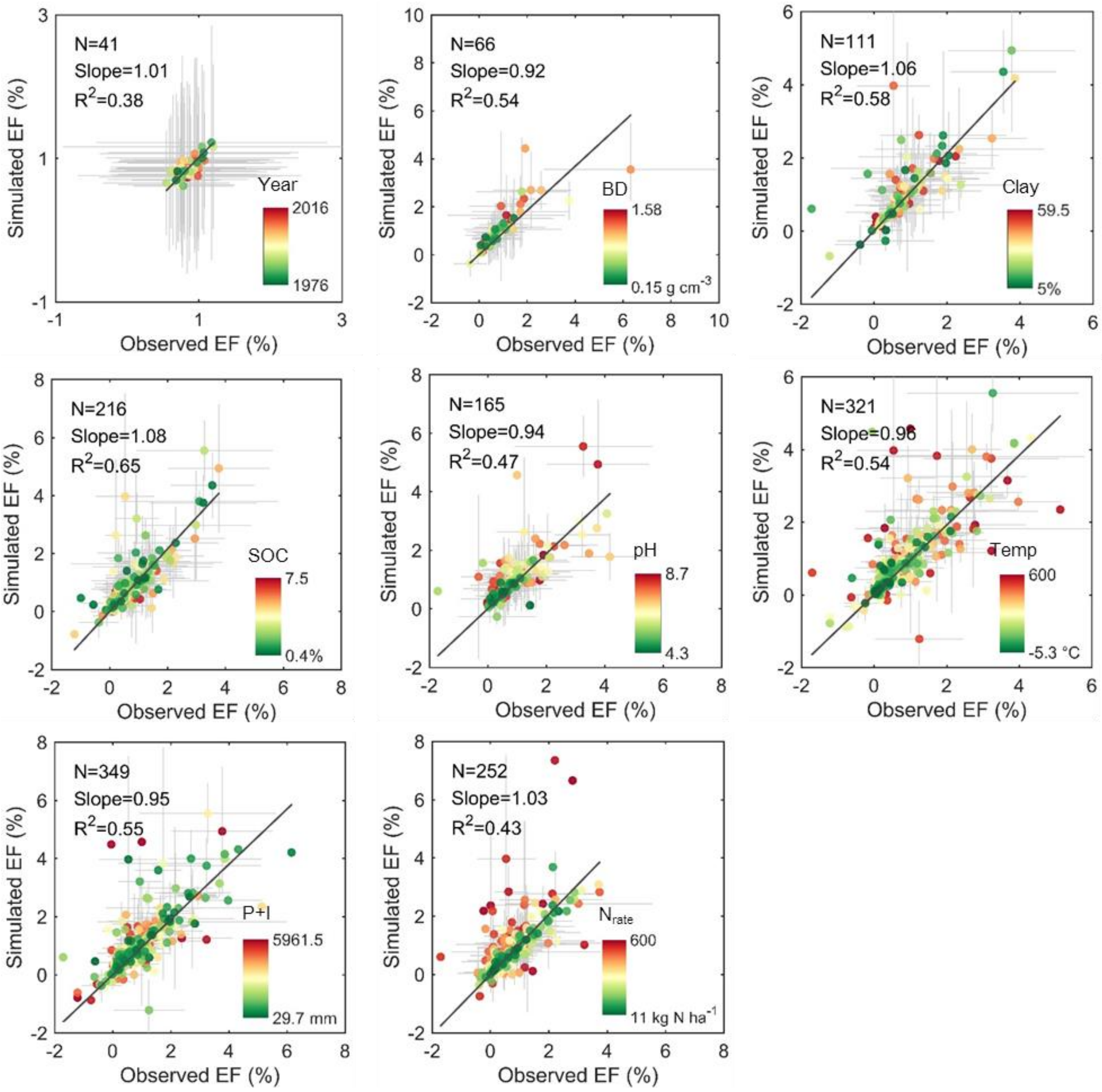

Figure S2. Model performance of interannual variability of EF and the sensitivity of EF to environmental changes. R^2 values before and after slash are for the means and SEs of E_{N2O} respectively.

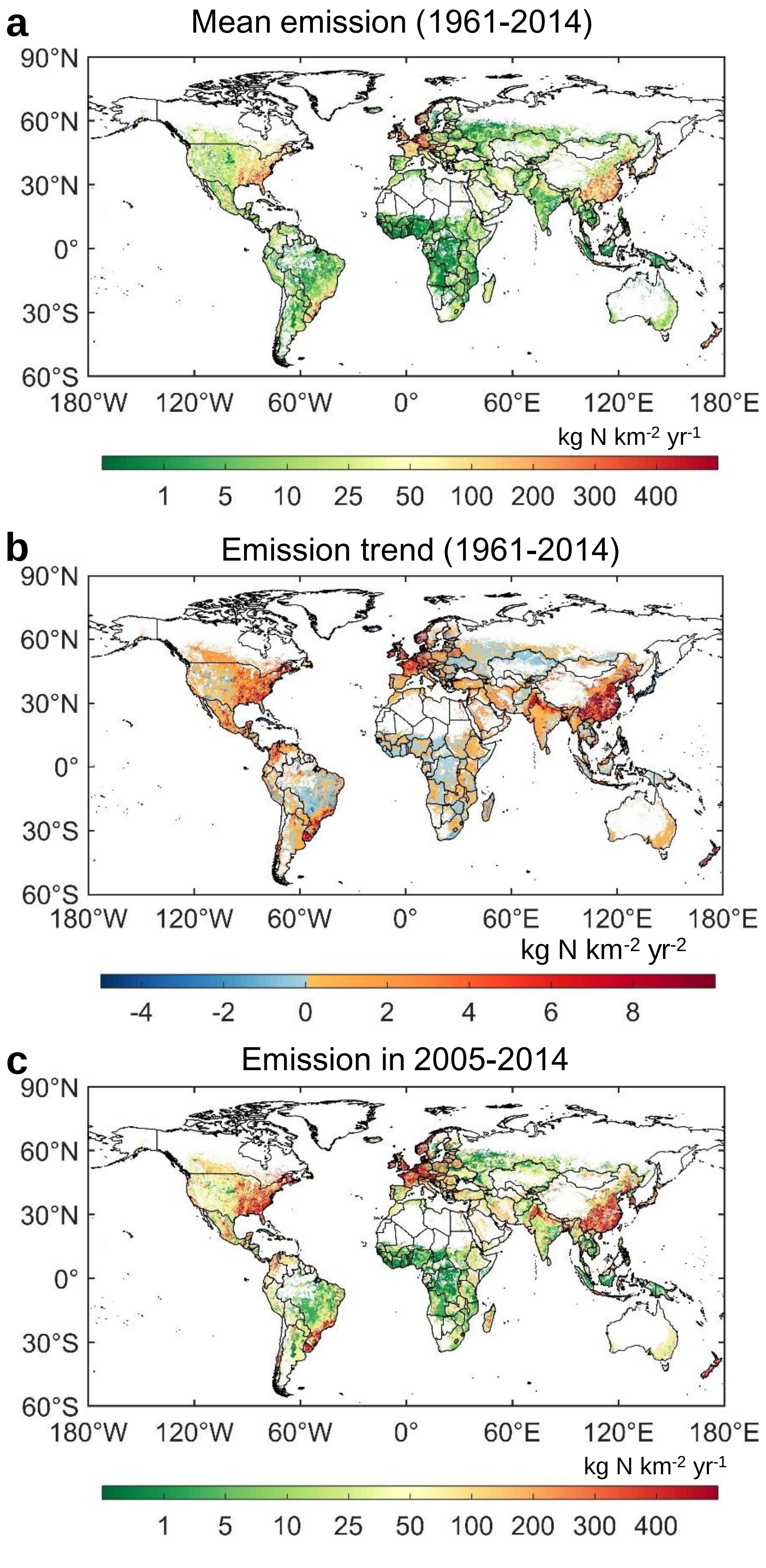

Figure S3. Spatial patterns of N_2O emission across global croplands by decade. a, mean emissions in 1961-2014, **b**, emission trend in 1961-20141, **c**, **h**, 2005-2014.

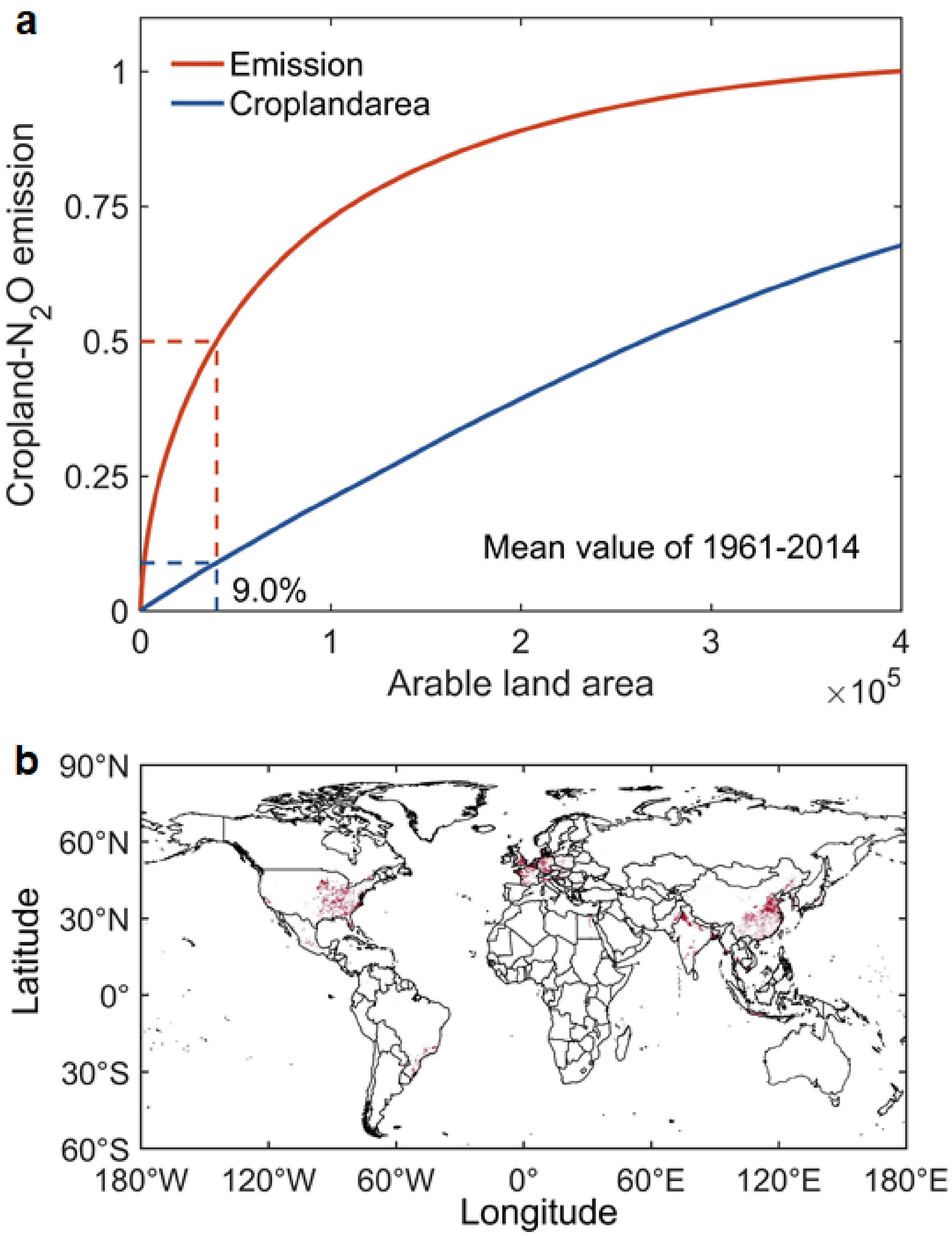

Figure S4. Cumulative proportions of cropland-N_2O emissions and arable land areas over the period 1961-2014. Approximately 50% of cumulative cropland-N_2O emissions originated from only 9.0% of global croplands over the period 1961-2014. The spatial pattern of croplands that contributed 50% of cumulative cropland-N_2O emissions is provided in panel **b**.

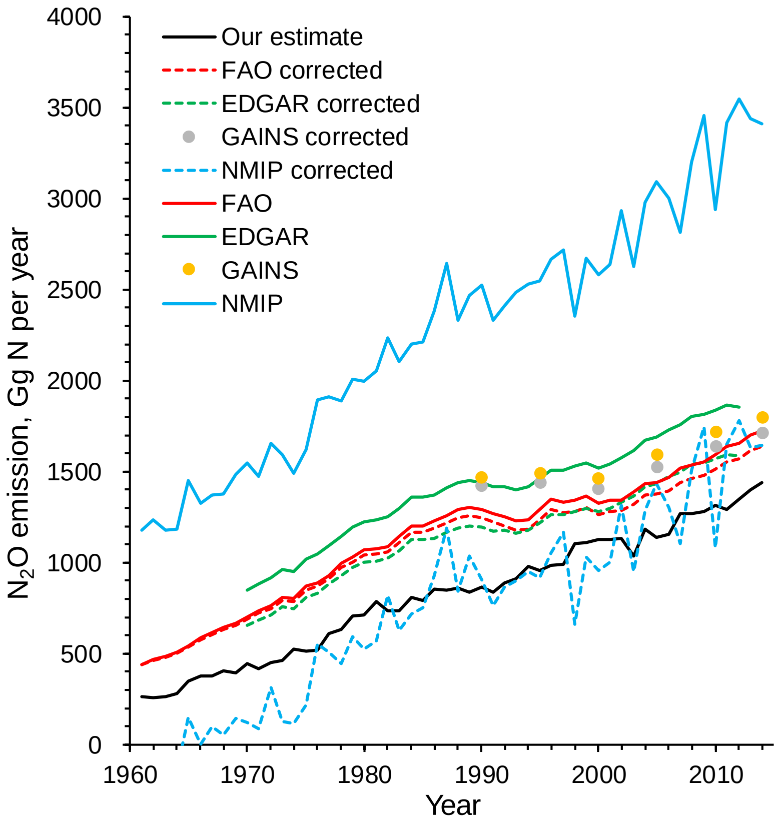

Figure S5. Temporal evolution of cropland-N_2O emissions. FAOSTAT: the Food and Agriculture Organization[9], EDGAR: the Emissions Database for Global Atmospheric Research version 4.3.2[10], GAINS: the Greenhouse Gas and Air Pollution Interactions and Synergies[11], NMIP: the N_2O Model Inter-comparison Project[12]. Note that solid and dash lines represent the values before and after correction. To compare with our estimate, FAOSTAT is corrected by removing the contributions from synthetic fertilizers applied to pasture. Similarly, EDGAR is corrected by removing the contributions from synthetic fertilizers applied to pasture and soil mineralization. NMIP is corrected by removing the contributions from 'background' emissions. Such 'background' emissions of N_2O flux was simulated as $E_{ijt}^{0} = \gamma_{ij}\left(x_k\right)$, where $x_k \in \Omega_i,\ \forall i$. This upscaling model were calibrated by using BRRT v2 against 469 observations for upland crops and 67 observations for paddy rice at the zero-N control sites from global cropland N_2O observation dataset. Cropland area was obtained from HYDE 3.2.1. In addition, we estimated 'background' emissions by multiplying the global mean N_2O fluxes from Kim et al.[7] (1.08 kg N ha^{-1} yr^{-1}) and Shcherbak et al.[1,7] (1.22 kg N ha^{-1} yr^{-1} for upland crops and 0.29 kg N ha^{-1} yr^{-1} for paddy rice) with global cropland area data from the FAO. It should be noted that these estimates of 'background' anthropogenic emissions did not consider the dynamics of soil N storage.

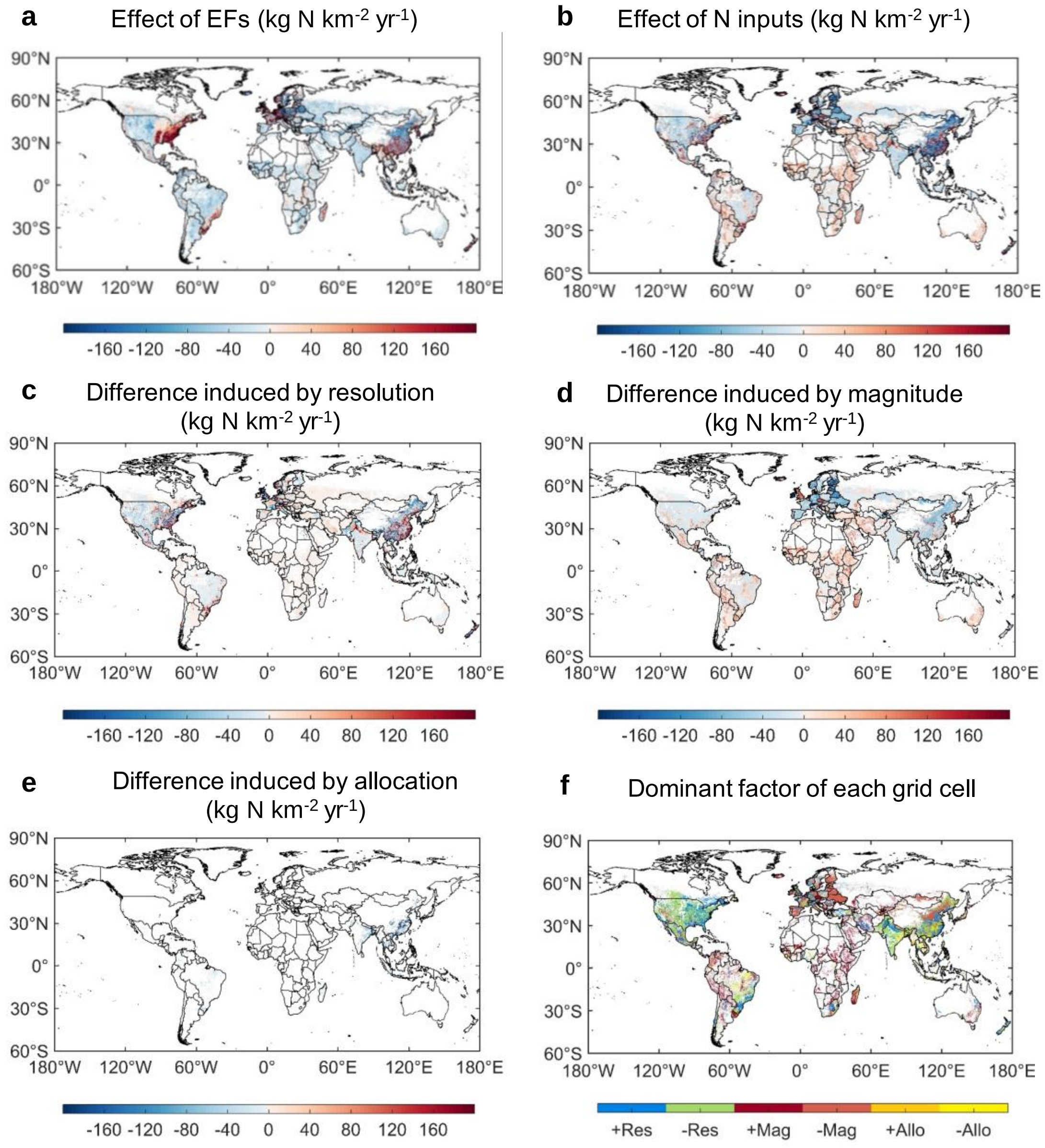

Figure S6. Spatial patterns of dominant drivers of the difference in cropland-N_2O emissions. **a**. Emission difference due to the effect of the updated EFs. **b**. Emission difference due to the effect of the revised N inputs. **c**. Emission difference due to the effect of the detailed spatial resolution of N inputs. **d**. Emission difference due to the effect of the magnitude of N inputs. **e**. Emission difference due to the effect of the crop-specific allocation of N inputs. **f**. Dominant factor of emission difference, defined as the driving factor that contributes the most to the difference in cropland-N_2O emissions in each copland grid cell. The driving factors include the updated EFs and the revised N inputs. A prefix '+' of the driving factors indicates a positive effect on emission difference, whereas '−' indicates a negative effect.

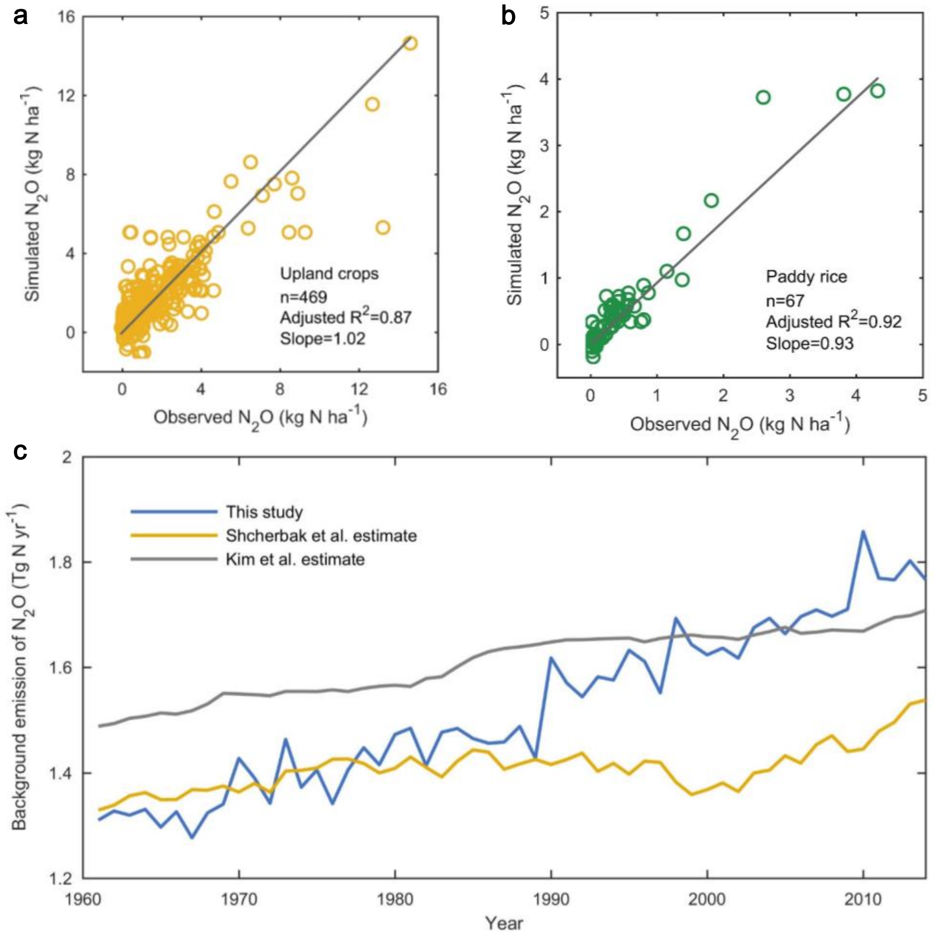

Figure S7. The estimate of global ‘background’ emissions of N_2O. a. Model performance for upland crops. **b.** Model performance for paddy rice. Note that n, adjusted R^2, and Slope denote the number of measurements, adjusted coefficient of determination, and slope of the regression line with the intercept set to zero, respectively. **c**. global ‘background’ emissions estimated based on our upscaling model and two previous studies. Note that the method for upscaling model can be found in the Methods. In addition, we estimated ‘background’ emissions by multiplying the global mean N_2O fluxes from Kim et al.[7] (1.08 kg N ha^{-1} yr^{-1}) and Shcherbak et al.[1,7] (1.22 kg N ha^{-1} yr^{-1} for upland crops and 0.29 kg N ha^{-1} yr^{-1} for paddy rice) with global cropland area data from the FAOSTAT.

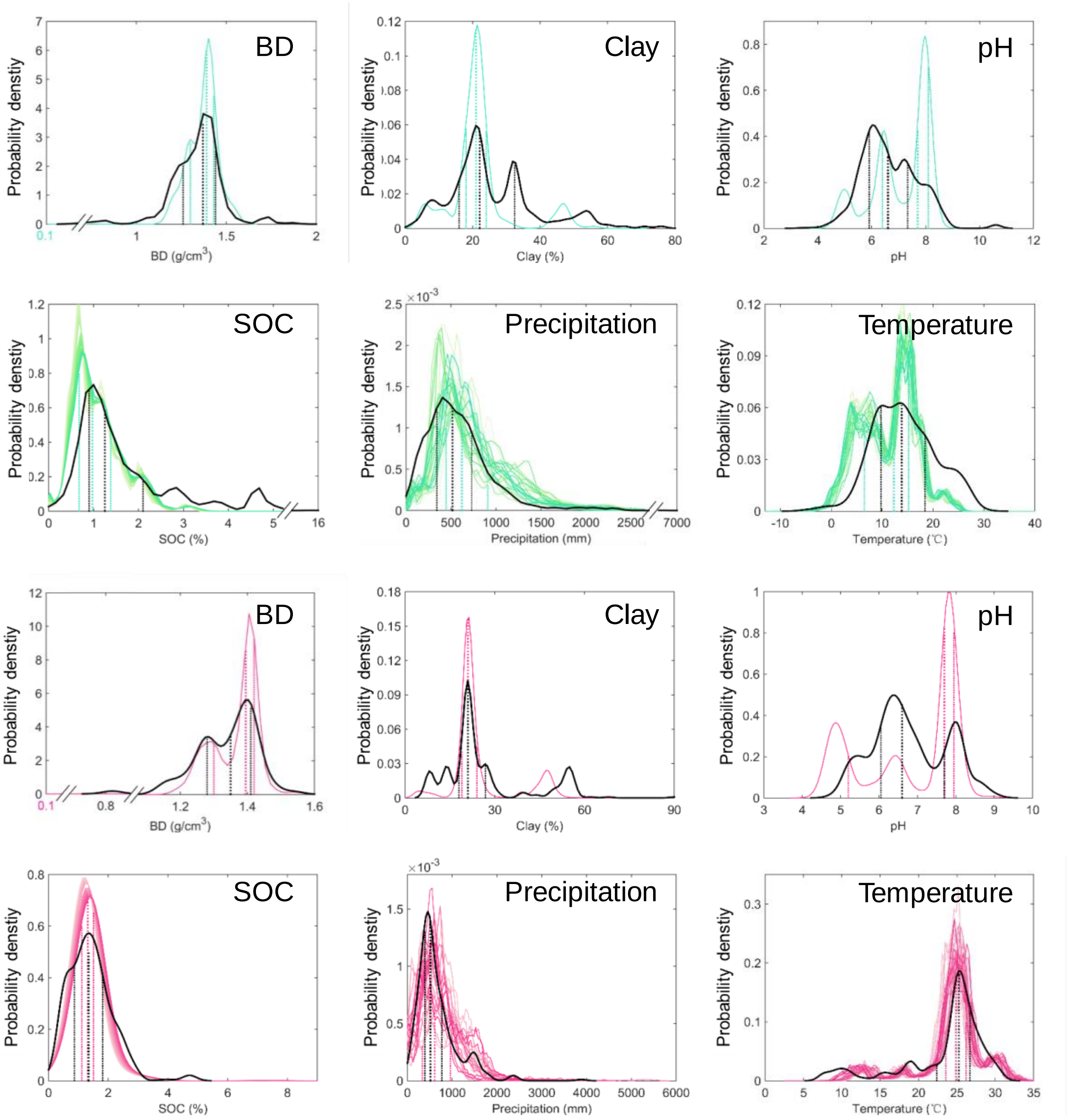

Figure S8. Comparison of probability density of soil and climate factors between our global cropland N_2O observation data (black) and global data over all croplands (green for upland crops and red for paddy rice). Global data of soil and climate factors are obtained from 1-km Harmonized World Soil Database (HWSD) v1.2 (http://www.iiasa.ac.at/) and CRU TS v. 3.23 (https://crudata.uea.ac.uk/cru/data/hrg/), respectively. Probabilities of 25%, 50% and 75% quantile are represented as three dash lines from left to right.

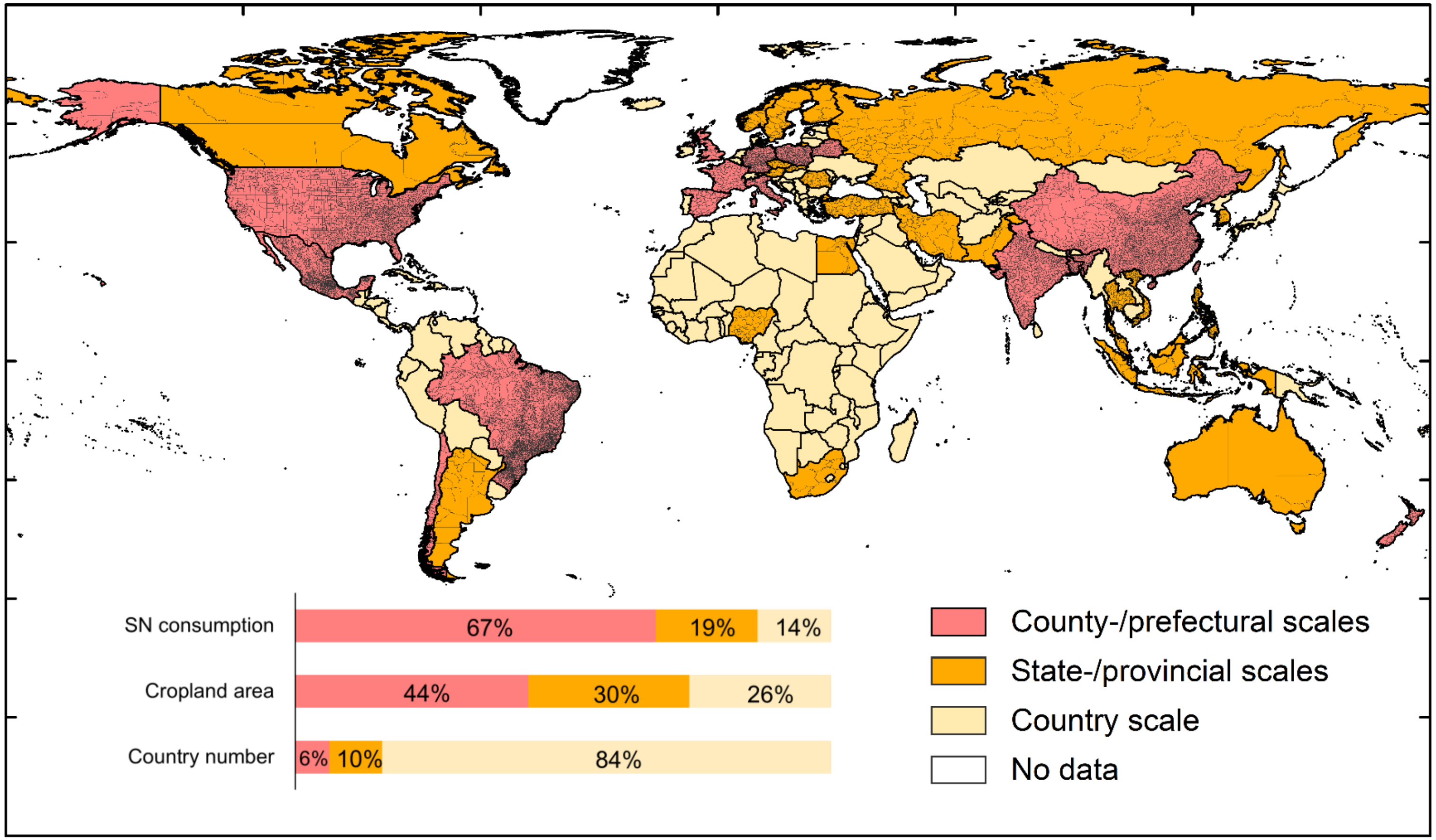

Figure S9. Spatial resolution of N-fertilizer consumption across 235 countries. Note: sub-national statistics (i.e., county-, municipal, provincial or state-levels) of N-fertilizer consumption were collected from 38 countries (colored as red and dark yellow), covering 15,593 administrative units (grey boundary); the national statistics of N-fertilizer consumption were acquired from the FAOSTAT for the other 197 countries in the world. The shares of sub-national statistics were calculated for the global total consumption, cropland area, and country number.

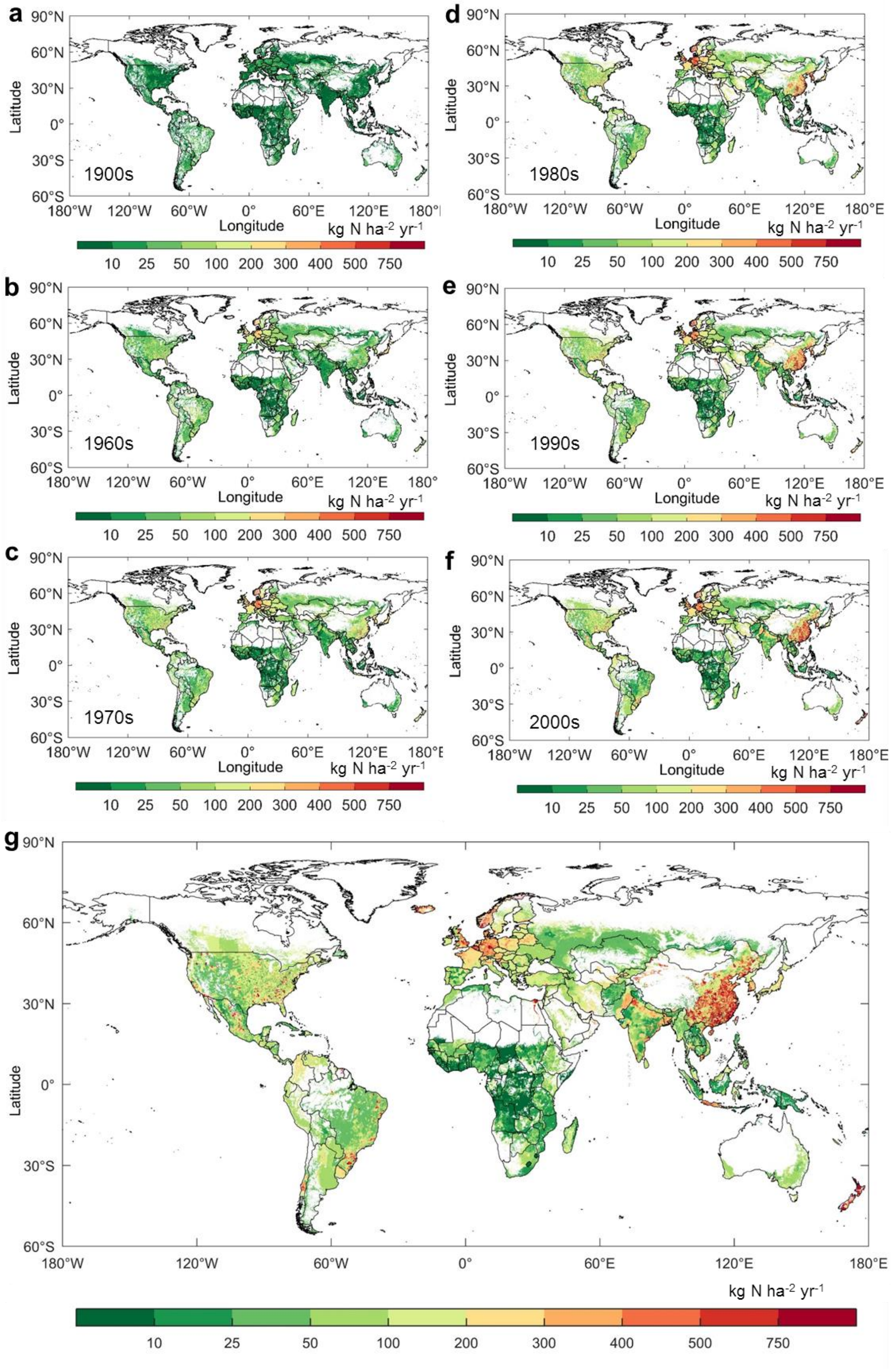

Figure S10. Five-minute grid maps of global N application rates in the 1900s (a), 1960s(b), 1970s (c), 1980s (d), 1990s (e), 2000s (f), 2005-2014 (g).

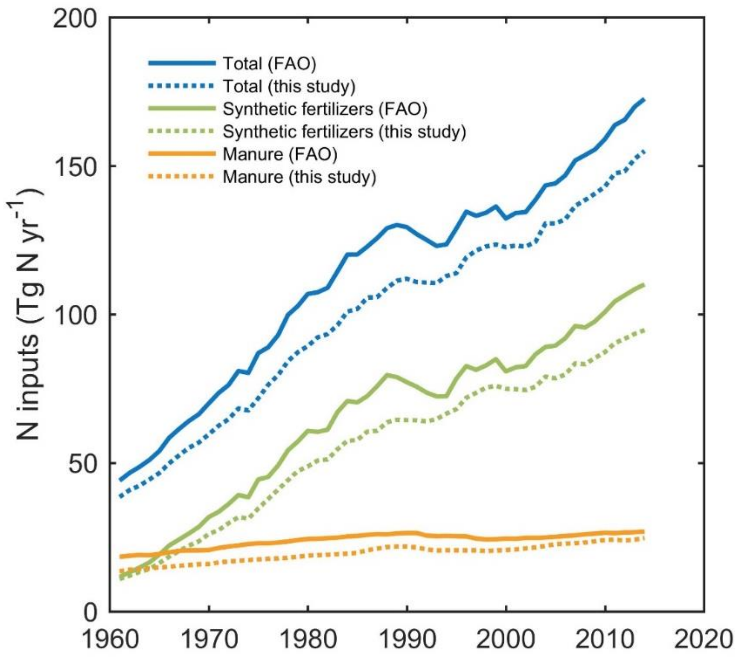

Figure S11. Comparison of global N inputs applied to croplands between our estimates and FAO. Total N inputs are the sum of synthetic fertilizers, livestock manure, and crop residues applied to croplands. Total N inputs of this study is aggregated from the sub-national statistics, while that of FAO is obtained from national data at http://www.fao.org/faostat/en/#data, where the pasture synthetic fertilizers application is removed.

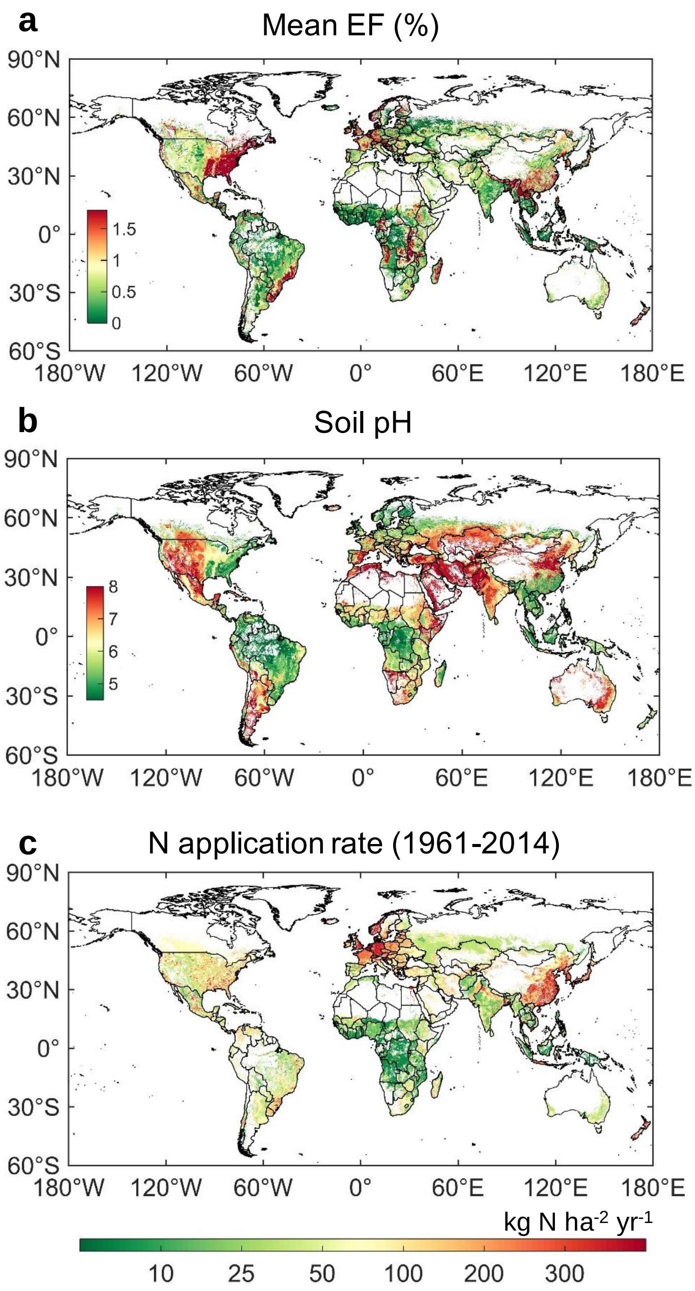

Figure S12. Spatial patterns of cropland-N_2O EFs, soil pH, and N application rate over the period 1961-2014.

References cited

1. Shcherbak I., Millar N., Robertson G. P.;Global metaanalysis of the nonlinear response of soil nitrous oxide (N2O) emissions to fertilizer nitrogen;*Proceedings of the National Academy of Sciences of the United States of America*;2014,111(25):9199-9204.

2. Ma L., Shan J., Yan X. Y.;Nitrite behavior accounts for the nitrous oxide peaks following fertilization in a fluvo-aquic soil;*Biology and Fertility of Soils*;2015,51(5):563-572.

3. Song Xiaotong, Liu Min, Ju Xiaotang, Gao Bing, Su Fang, Chen Xinping, Rees Robert M.;Nitrous Oxide Emissions Increase Exponentially When Optimum Nitrogen Fertilizer Rates Are Exceeded in the North China Plain;*Environmental Science & Technology*;2018,52(21):12504-12513.

4. Food and Agricultural Organization of the United nations (FAO), FAOSTAT Emissions Database, Agriculture, Synthetic Fertilizers Retrieved 2017, from http://www.fao.org/faostat/en/#data/GY.

5. Akiyama H., Yagi K., Yan X. Y.;Direct N2O emissions from rice paddy fields: Summary of available data;*Global Biogeochemical Cycles*;2005,19(1).

6. Stehfest E., Bouwman L.;N2O and NO emission from agricultural fields and soils under natural vegetation: summarizing available measurement data and modeling of global annual emissions;*Nutrient Cycling in Agroecosystems*;2006,74(3):207-228.

7. Kim D. G., Giltrap D., Hernandez-Ramirez G.;Background nitrous oxide emissions in agricultural and natural lands: a meta-analysis;*Plant and Soil*;2013,373(1-2):17-30.

8. Kim D. G., Hernandez-Ramirez G., Giltrap D.;Linear and nonlinear dependency of direct nitrous oxide emissions on fertilizer nitrogen input: A meta-analysis;*Agriculture Ecosystems & Environment*;2013,168:53-65.

9. Food and Agricultural Organization of the United nations (FAO). FAOSTAT data. Retrieved June 18, 2018, from http://www.fao.org/faostat/en/#data.

10. Janssens-Maenhout G., Crippa M., Guizzardi D., Muntean M., Schaaf E., Dentener F., Bergamaschi P., Pagliari V., Olivier J., Peters J., van Aardenne J., Monni S., Doering U., Petrescu R., Solazzo E., Oreggioni G.;EDGAR v4.3.2 Global Atlas of the three major Greenhouse Gas Emissions for the period 1970-2012;*Earth Syst Sci Data Discuss*;2019,2019:1-52.

11. Winiwarter W., Hoglund-Isaksson L., Klimont Z., Schoopp W., Amann M.;Technical opportunities to reduce global anthropogenic emissions of nitrous oxide;*Environmental Research Letters*;2018,13(1).

12. Tian Hanqin, Yang Jia, Xu Rongting, Lu Chaoqun, Canadell Josep G., Davidson Eric A., Jackson Robert B., Arneth Almut, Chang Jinfeng, Ciais Philippe, Gerber Stefan, Ito Akihiko, Joos Fortunat, Lienert Sebastian, Messina Palmira, Olin Stefan, Pan Shufen, Peng Changhui, Saikawa Eri, Thompson Rona L., Vuichard Nicolas, Winiwarter Wilfried, Zaehle Sönke, Zhang Bowen;Global soil nitrous oxide emissions since the preindustrial era estimated by an ensemble of terrestrial biosphere models: Magnitude, attribution, and uncertainty;*Global Change Biology*;2019,25(2):640-659.
